# Congenital Tufting Enteropathy, a Rare Cause of Diarrhea and Malnourishment in Arab Child with Genetic and Histopathology Investigations

**DOI:** 10.1155/2023/6301065

**Published:** 2023-01-25

**Authors:** Shooq Alkhalifa, Aysha Darwish, Mohamed Awadh, Salman M. Alkhalifa, Abdulla Darwish

**Affiliations:** ^1^BDF Royal Medical Services, Riffa, Bahrain; ^2^RCSI-Bahrain, Riffa, Governorate, Bahrain

## Abstract

Congenital tufting enteropathy (CTE), also known as intestinal epithelial dysplasia (IED), is a rare autosomal recessive disorder due to EPCAM gene mutation. It is a rare congenital enteropathy that presents in early infancy as an intractable diarrhea that is independent of breast formula feeding that requires life-long total parental nutrition (TPN) to acquire adequate calories and fluid intake or small bowel transplantation in severe cases. Here, we report a case of intestinal failure due to congenital tufting enteropathy in a 3-year-old girl who presented with loose stools and failure to thrive. This study aims to review the literature about CTE and discuss the clinicopathological aspects and to be able to distinguish it from other causes of congenital diarrheal disorders (CDDs).

## 1. Introduction

Congenital tufting enteropathy (CTE) is a rare autosomal recessive disease and an unusual cause of chronic watery diarrhea and failure to thrive in infancy with some congenital features observed in the syndromic form [1, 2]. Mutations in certain genes attributed to the CTE such as EPCAM and SPINT2 genes have been discussed thoroughly in the literature [3–5].

Incidence of CTE is estimated to be 1 in 50,000–100,000 live births in Western Europe and a higher incidence in Middle Eastern Families mainly due to high degrees of consanguinity [6, 7]. The diagnosis of CTE can be determined by histological examination of the duodenal biopsy which shows a characteristic epithelial tufting. The aim of this report is to present a case of CTE in a three-year-old girl who presented with persistent diarrhea and failure to thrive with a review of the literature.

## 2. Case Report

A three-year-old Arab girl, a known case of congenital tufting enteropathy (CTE), presented to the pediatric clinic complaining of diarrhea, delayed growth, and failure to gain weight. Diagnosis of CTE was confirmed at the age of one in Jordan, confirmed by molecular genetic testing which showed homozygosity for EPCAM c.499 dup. The patient is currently suffering from intestinal failure due to CTE and was admitted to our hospital to continue her total parental nutrition (TPN) therapy.

The medical history showed that the patient was admitted in Jordan at the age of 6 months, and during her stay, she had a recurrent staph epidermidis infection, cholestasis, and gastroesophageal reflux disease (GERD) secondary to hiatal hernia and recurrent vomiting resolved after starting her on Neocate formula. She had a bone marrow aplastic crisis due to a viral infection which resolved spontaneously. Family history revealed a consanguineous marriage as well as a sibling with the same disease who died at the age of five years due to sepsis from the indwelling catheter.

**Figure 1:**
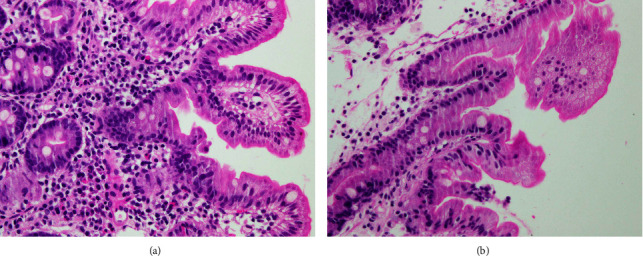
(a) and (b) Duodenal biopsy shows the presence of focal crowding resembling tufts; it contains closely packed enterocytes with clusters of round, teardrop-shaped projections in the apical cytoplasm (H&E stain x400).

Her admission weight was 9 kg, her length was 80 cm, and BMI was 13.4 which is below the 10^th^ percentile according to her age. Laboratory investigations showed ALT: 12 IU\L, AST: 33 IU\L, GGT: 12 IU\L, total bilirubin: 6.5 mmol\l (direct bilirubin: 2.9 mmol\L), CBC, and electrolytes were normal. She underwent esophagogastroduodenoscopy and colonoscopy during which mucosal biopsies are taken at multiple sites. Histological microscopic examination of the duodenum showed variable villous shortening and atrophy with focal areas showing disorganized crowded surface epithelium with extrusion of apical tufting consistent with tufting enteropathy (Figures 1(a) and 1(b)). Gastric biopsy showed gastritis and esophageal biopsy was within normal limits.

During her hospital stay, her main issues were as follows:

1. Flushing and abdominal pain with lipid infusion as this was noted repeatedly; however, these symptoms were limited after changing the infusion time and type of lipids.4
2. Stagnant growth despite being on aminoplasma, lipids, and glucose.
3. Solid food feeding aversion as she refuses solid foods; the patient was assessed by the speech and swallowing pathologist and found not to have any issues with choking or gagging.
4. Bone marrow suppression with cytopenia as she was started on broad-spectrum antibiotics and full septic workup cultures were negative; however, serology for EBV was positive and her condition resolved spontaneously after one week.
5. *Clostridium difficile* colitis which was treated with oral vancomycin.

She is still in the hospital receiving her TPN therapy.

## 3. Discussion

CTE patients have abnormal development and differentiation of enterocytes, which has been linked to mutation of the EPCAM gene (2p21) in 73% of cases or mutations in the SPINT2 gene (19q13.2) in 21% of cases [3–5]. In rare cases, no mutations in the EPCAM or SPINT2 gene were found [8]. EpCAM protein is expressed on most normal epithelial cells surfaces and functions as a cell adhesion molecule. EpCAM mutations can disrupt the association with *α*-actin, claudin-7, or E-cadherin, causing disturbance of mucosal integrity and eventual formation of the typical tufts seen in CTE [3–5]. SPINT2 protein, also called hepatocyte growth factor activator inhibitor type 2 (HIA-2), is a transmembrane protein thought to be involved in epithelial regeneration, as well as in the signaling pathways of NF-*κ*B and TGF-*β*. It was suggested that HAI-2-carrying CTE missense mutations are less efficient in inhibiting matriptase than wild-type (WT) HAI-2, resulting in decreased cellular levels of EpCAM [5].

CTE is characterized by heterogeneous clinical and histological findings which vary depending on the degree of mutation. Patients could present with gastrointestinal symptoms such as vomiting, diarrhea or abdominal distention, or atypical presentation that is known as the syndromic form of CTE (SCTE). These features include ophthalmologic signs such as photophobia, corneal erosion, cataract, and superficial punctuated keratitis (SPK) and as well as atresias such as choanal atresia or anal atresia [8–11].

Histopathological examination findings are villous atrophy of the intestinal epithelium, tufts formation, crypt hyperplasia, minimal lymphocytosis in the lamina propria, and increased desmoglein staining of the epithelial cell membrane [12]. Sometimes, such characteristic tufts could be absent in the biopsy of early CTE patients with typical clinical symptoms, while the absence of EpCAM in the epithelium could be confirmed by further immunohistochemically staining for MOC-31 [13].

The differential diagnosis includes microvillus inclusion disease, which can show villous atrophy and scant inflammation, but lacks surface tufting and demonstrates a characteristic apical cytoplasmic inclusion ultrastructurally in duodenal enterocytes [14]. The other differentials are congenital chloride diarrhea, congenital sodium diarrhea, and glucose-galactose malabsorption, which are diagnosed based on the clinical setting [11].

Management of CTE should be based on early detection and appropriate total parental nutrition (TPN) therapy; it can also cause irreversible intestinal failure (IF) and therefore requires early detection and analysis of irreversible risk, while some patients with a milder phenotype will require partial TPN ranging from 3 to 6 times per week [15]. It is always important to carefully monitor any case on TPN to ensure appropriate growth. In severe cases, intestinal transplantation is required with careful selection of the proper candidates [16, 17].

It is important to early recognize CTE as it has high morbidity and mortality, especially as the symptoms may not be specific and overlap with other conditions; therefore, it should be considered in the differential diagnosis in young children presented with chronic gastrointestinal symptoms when accompanied with congenital malformations and a family history of congenital diarrheal disorders (CDDs) which will aid in early detection, hence improving the patient outcome and growth by enabling CDD specific treatment. Awareness of this rare entity is also important for the histopathologist as it can be easily overlooked; careful microscopic examination of the villi surface must be performed especially in young children presented with chronic diarrhea and failure to thrive.

## 4. Conclusion

Congenital tufting enteropathy is a rare congenital abnormality of the intestinal mucosa that results in severe malnutrition and growth retardation leading to significant morbidity and mortality. Diagnosis of CTE is based on clinical, histological, and molecular genetic testing in such patients and by exclusion of other causes of congenital and acquired causes of severe diarrheal disorders in children.
